# Levels of anti-CMV antibodies are modulated by the frequency and intensity of virus reactivations in kidney transplant patients

**DOI:** 10.1371/journal.pone.0194789

**Published:** 2018-04-11

**Authors:** María Iglesias-Escudero, Marco Antonio Moro-García, Raquel Marcos-Fernández, Alejandra García-Torre, Marta Elena Álvarez-Argüelles, María Luisa Suárez-Fernández, Pablo Martínez-Camblor, Minerva Rodríguez, Rebeca Alonso-Arias

**Affiliations:** 1 Immunology Department, Hospital Universitario Central de Asturias (HUCA), Oviedo, Spain; 2 Department of Microbiology, HUCA, Oviedo, Spain; 3 Nephrology Department, HUCA, Oviedo, Spain; 4 Statistics Department, HUCA, Oviedo, Spain; University of San Francisco, UNITED STATES

## Abstract

Anti-CMV (cytomegalovirus) antibody titers are related to immune alterations and increased risk of mortality. To test whether they represent a marker of infection history, we analyzed the effect of viral reactivations on the production of specific antibodies in kidney transplant patients. We quantified CMV-DNAemia and antibody titers in 58 kidney transplant patients before transplantation and during a follow-up of 315 days (standard deviation, SD: 134.5 days). In order to calculate the intensity of the infection, we plotted the follow-up time of the infection on the x-axis and the number of DNA-CMV copies on the y-axis and calculated the area under the curve (CMV-AUC). The degree of T-lymphocyte differentiation was analyzed with flow cytometry, the cells were labelled with different monoclonal antibodies in order to distinguish their differentiation state, from naive T-cells to senescent T-cells. Peak viremia was significantly higher in patients experiencing a primary infection (VI) compared to patients experiencing viral reactivation (VR). Our data indicate that the overall CMV viral load over the course of a primary infection is significantly higher than in a reactivation of a previously established infection. Whereas patients who experienced an episode of CMV reactivation during the course of our observation showed increased levels of CMV-specific antibodies, patients who did not experience CMV reactivation (WVR) showed a drop in CMV antibody levels that corresponds to an overall drop in antibody levels, probably due to the continuing immunosuppression after the renal transplant. We found a positive correlation between the CMV viremia over the course of the infection or reactivation and the CMV-specific antibody titers in the examined patients. We also observed a positive correlation between anti-CMV titers and T-cell differentiation. In conclusion, our data show that anti-CMV antibody titers are related to the course of CMV infection in kidney transplant patients.

## Introduction

After primary infection, CMV establishes chronic infections mainly in the myeloid cell compartment and endothelial cells. Virus reactivation from latency can be caused by various situations, such as inflammation, infection, stress, or immunosuppression [1, 2]. Humoral immunity is considered effective in restricting viral dissemination and limiting the severity of the disease [3]. Meanwhile, the predominant adaptive immune response to the virus is thought to be mediated by T-lymphocytes. The CMV-specific humoral and cellular immunity is not able to eliminate the virus, which periodically reactivates. Immunological surveillance is the main regulator of the clinical presentation of active CMV infection [4, 5]. Thus, although reactivations from latency are likely to occur in immunocompetent hosts, their functional immune system avoids the spread of the virus, and the infection is essentially asymptomatic. However, in immunosuppressed individuals, reactivations may have serious clinical consequences. Transplant recipients receiving immunosuppressive therapy are at an increased risk of active CMV infection and disease. As a result, despite the high efficacy of current antiviral preventive strategies, CMV infection is an important complication in patients receiving solid organ transplants, including kidney recipients [6–8]. The risk is also affected by the serostatus of the donor and recipient, with transplantation of a seropositive organ to a seronegative recipient placing the recipient at higher risk of CMV disease [9]. The course of CMV infection influences both the graft and also the patient survival [10] through direct and indirect mechanisms. Indirect effects have been associated with acute and chronic allograft rejection events, as well as new onset diabetes and accelerated coronary artery atherosclerosis in transplant patients [11].

In immunocompetent individuals, CMV is considered a marker of immune dysfunction, and an etiologic agent for chronic diseases. CMV infection has been related to situations with a high inflammatory component, such as cardiovascular diseases, cancers and cognitive and functional disorders [12–15]. It has been reported, that increased risk of mortality is not only related to CMV seropositivity, but it also appears to be positively correlated with CMV-specific antibody levels, probably through the connection between chronic CMV infection and cardiovascular disease [16–19]. When the CMV virus is reactivated, there is an increase in proinflammatory molecules, such as IL-6, tumor necrosis factor alpha (TNF-α) and C-reactive protein (CRP) [20]. The induction of high levels of CMV-specific T-lymphocytes may occur due to a large number of virus reactivations or an enhanced response to the virus, but ultimately a substantial proportion of the immune repertoire may be required to control replication of the virus. CMV seropositivity is associated with the phenomenon of immunosenescence, which is characterized by the clonal expansion of T-cells with a highly differentiated phenotype and the acquisition of new functional features, and a decrease in the individuals’ ability to resist new infections [21–24]. We recently demonstrated that titers of anti-CMV antibodies are related to the differentiation status of T-lymphocytes and immunocompetence in the elderly [25]. Indeed, despite evidence suggesting that CMV induces the aging of T-lymphocytes, more frequent and/or intense reactivations may also be a consequence, and not just a cause, of impaired immunological responses because CMV can suddenly reactivate after periods of immunosuppression. The quantification of CMV reactivations may be a measure of the individual’s immunocompetence, and titers of anti-CMV antibodies may be indicative of the number and intensity of the reactivations.

High levels of anti-CMV antibodies are thought to represent more frequent virus reactivations, although this correlation has not been conclusively shown. Transplant patients enable us to study the course of CMV infection during a short time period and analyze whether variations in anti-CMV antibody levels reflect the intensity and frequency of virus reactivations in these immunosuppressed patients.

## Material and methods

Fifty-eight transplant patients (23 females and 35 males) who received a renal allograft in the Hospital Universitario Central de Asturias (Oviedo, Spain) were enrolled in the study. Patients with signs of rejection (biopsy-proven acute rejection) during the follow-up were not included in the study. The participant recruitment dates ranged between April 2013 and June 2016. The mean follow-up time was 315 days (SD: 134.5 days). Clinical features of the kidney transplant patients are shown in Table 1. Participant’s informed written consent was obtained prior to participating in the study. The study was approved by the Hospital Central de Asturias (Oviedo, Spain) ethics committee.

**Table 1:** Characteristics of the study subjects.

Clinical features	N = 58
**Age (years)**	
**Mean ± SE**	53.3 ± 13.2
**Range**	36–76
**Follow up (days)**	315 ± 134.5
**Gender**	
Male	35 (60.3%)
Female	23 (39.6%)
**Underlying disease**	
Renal vascular disease	29 (50%)
Polycystic kidney disease	11 (18.9%)
Alport syndrome	3 (5.2%)
Others	13 (22.3%)
**CMV serological status**	
**CMV+**	42 (72.4%)
VR (With reactivations)	26 (61.9%)
WVR (Without reactivations)	16 (38.1%)
**CMV-**	16 (27.6%)
VI (Recently infected)	7 (12.1%)
WVI (Not infected)	9 (15.5%)
**Induction treatments**	
ATG	9 (15.5%)
Basilisimab	22 (37.9%)
Both	2 (3.4%)
None	25 (43.1%)
**Calcineurin inhibitor**	
Tacrolimus	56 (96.6%)
Cyclosporine	2 (3.4%)

### CMV prophylaxis and treatment of CMV disease

All the patients who were CMV-seropositive and received induction treatment with basiliximab or timoglobulin or CMV-seronegative and received an organ from a CMV-seropositive donor, received prophylactic treatment with oral valganciclovir (900 mg/24 h adjusted for renal function) for 100 days. All the patients claimed to have rigorously followed the prophylactic treatment for CMV.

In all the patients, CMV-DNA copies were periodically monitored using real-time polymerase chain reaction (PCR) in whole blood (first month, once a week; second to third months, once every two weeks; fourth to twelfth months, once a month, and after the first year, once a year or in the presence of symptoms). If CMV-DNA was detected (> 500 copies/ml), infection was considered positive, and a new quantification was performed 72 hours later, and then once a week until CMV-DNA was no longer detected. Treatment of CMV infection and disease consisted of oral valganciclovir (900 mg/12 h adjusted for renal function) for 15 days or until viremia became negative. If CMV infection or disease did not respond to oral treatment, intravenous ganciclovir (5 mg/kg every 12 hours, adjusted for renal function) was administered until all signs and symptoms of CMV disease had resolved and results of the CMV-DNA PCR had become negative.

### CMV serology

Levels of CMV-specific IgG antibodies were determined by the Vir-Enzyme-Linked ImmunoSorbent Assay (ELISA) anti-CMV IgG assay (Viro-Immun Labor-Diagnostika GmbH, Oberursel, Germany), carried out in accordance with the manufacturer’s specifications. The CMV antibodies are specifics against the pp65 molecule. CMV serostatus was interpreted by calculating the ratio: Cutoff Index = optical density (OD) value of sample / cutoff value, whereby a ratio of 1.0 is equivalent to the cutoff value. Cutoff indexes > 1.1 were considered positive. Quantification of anti-CMV antibody titers was performed through semi-quantitative titer calculation. Samples from each patient were always tested in the same assay.

### Quantitative CMV-PCR

Extraction of DNA from whole blood was followed by amplification and quantification using real-time PCR (Roche Diagnostics). A standard curve of known concentrations of target DNA was generated for each assay. Specific forward and reverse primers were 5´-GACTTCAGGGTACTGGAACTTTACT-3´ and 5´-ATTCGCGCATGATCTCTTCGA-3´, respectively. The selected probe was labelled FAM: 5´-AACGCAGCTCTTTCTG-3´. PCR consisted of 2 min at 50°C and then, 10 min at 95°C followed by 50 cycles of 15 s at 95°C and 1 min at 60°C each. The assays were performed using the LightCycler 480 II Real-Time PCR Instrument (Roche Diagnostics GmbH, Mannheim, Germany), and amplification data were analyzed using the LightCycler 480 Software (Roche Diagnostics). Results are expressed in CMV-DNA copies/ml plasma with a reportable range of 500–100,000 CMV-DNA copies/ml.

### Anti-HLA antibody detection

HLA antibodies were identified by LABScreen® assays (One-Lambda) using Luminex xMAP technology (Luminex Corp., Austin, TX, USA), following the manufacturer’s specifications. Patients’s serum samples were tested against HLA alleles using the LABScreen® Mixed kit for general screening. Positive sera were re-tested using the LABScreen® HLA Single Antigen to measure the specificity of the antibodies. The fluorescent signal for each HLA allele-coated bead (MFI: median fluorescence intensity) was measured using LABScan 100TM Flow Cytometry and analyzed by HLA-FusionTM software (One-Lambda). In all cases, anti-HLA antibodies detected with this technique were of the IgG isotype.

### Immunological phenotyping

For flow cytometry analysis, peripheral blood cells were surface-stained with anti-CD3 (fluorescein isotiocyanate, FITC) (BD Biosciences), anti-CD8 (phycoeritrin, PE), anti-CD4 (peridinin chlorophyll-A protein, PerCP), (Immunostep, Salamanca, Spain), anti-CD28 (allophycocyanin hilite, 7APC-H7), and anti-CD27 (phycoerythrin-Cyanine 7, PE-Cy). One hundred microliters of whole blood from each patient were stained with different combinations of labeled monoclonal antibodies for 20 min at room temperature. Samples were red-cell lysed with the FACS Lysing Solution (BD Biosciences), washed in phosphate buffered saline (PBS), and analyzed using FacsDiva software in a FACSCanto Cytometer (BD Biosciences). Appropriate isotype control monoclonal antibodies were used for marker settings.

### Statistical analysis

The results are expressed as the median and interquartile range (IR) or the mean and SD. Groups were compared using the non-parametric Mann-Whitney U test (for non-normally distributed data) or Student’s t test (for normally distributed data). Paired analyses were performed using the Wilcoxon non-parametric method when data were not normally distributed, or Student´s t test for paired samples. The outlier values were calculated by adding 1.5 times the IR to the 75th percentile. Correlations between variables were assessed using the non-parametric Spearman test (ρ), and multiple linear regression was used to consider the variables simultaneously. Analyses were performed using the SPSS 17.0 statistical software package program (SPSS Inc. Chicago, IL) and p-values of 0.05 or less were considered significant.

## Results

### The course of CMV infection during the follow-up period

The clinical features of the kidney transplant patients are shown in Table 1. At the moment of the transplant, 42 patients tested CMV-seropositive, and 16 patients were CMV-seronegative. Of the 16 CMV- patients transplanted, 10 received an organ from a CMV+ donor, and of them, 7 transplanted patients became infected. None of the CMV- patients who received an organ from a CMV- donor became infected. Transplanted patients were followed-up fora mean time of 315 days (SD: 134.5 days) and CMV-DNA quantification was performed a mean of 22.4 times per patient (SD: 10.25). During this time, 26 out of 42 CMV-seropositive patients suffered virus reactivations or re-infections, as evidenced by the detection of viral DNA. Seven out of 16 CMV-seronegative patients were virus infected (i.e. viral DNA was detected in these patients); however, only four of these patients displayed anti-CMV antibodies above the ELISA detection limit. The average number of points investigated in the patients included in the study was 27 (27±7 points). In patients who became infected or experienced a reactivation of the disease, the mean time from transplant to the detection of circulating virus DNA was 133 days (133±83 days).

**Fig 1:**
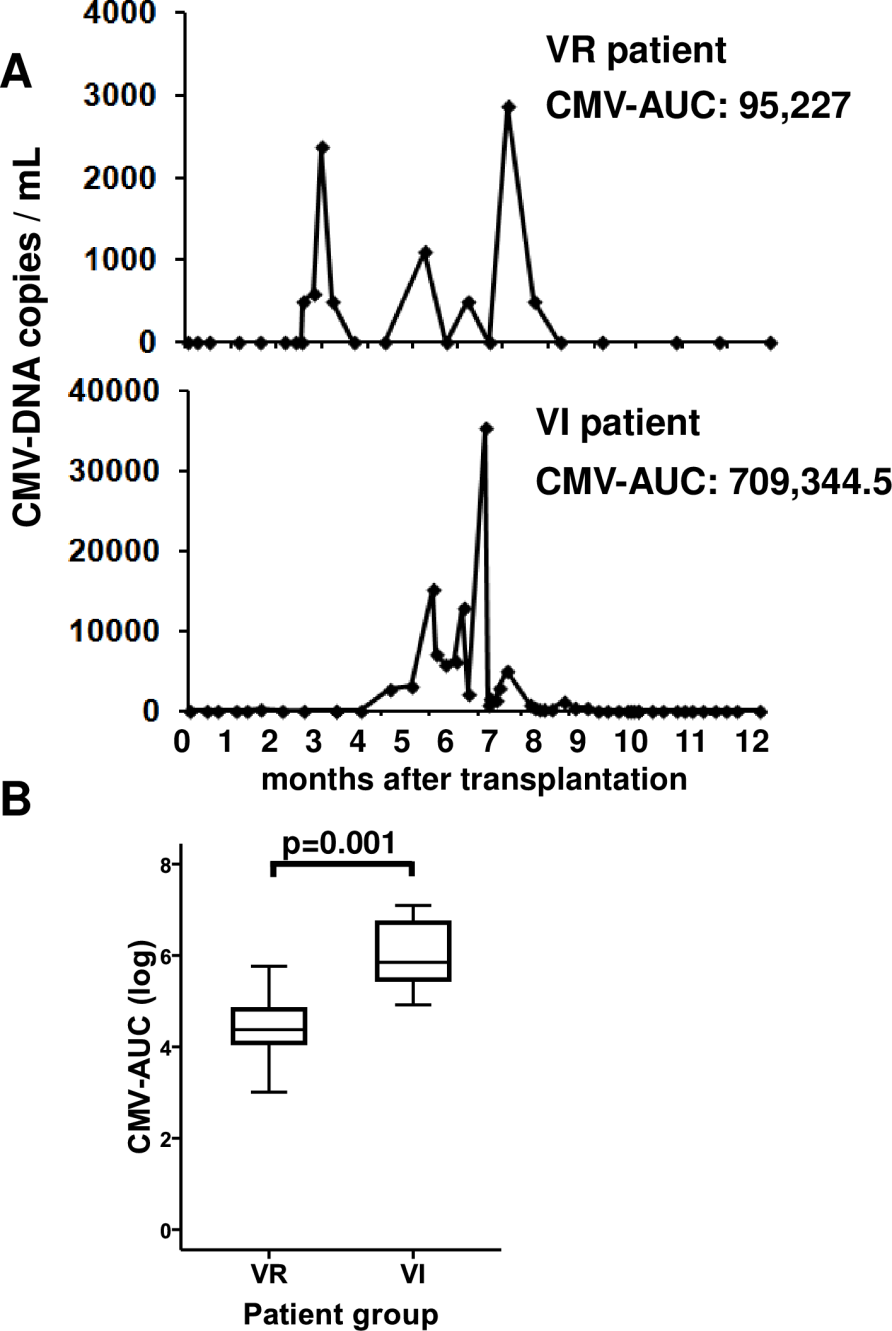
CMV-DNA copy number in VR and VI patients during the follow-up period after kidney transplantation. (A) Follow-up time and CMV-DNA copy number (copies/ml) are shown for representative VR and VI patients by calculating the CMV-AUC as a measure of infection intensity. (B) CMV-AUC in VR patients (n = 26 out of 42 CMV-seropositive patients) and VI patients (n = 7 out of 16 seronegative patients who were infected and subsequently became CMV-seropositive) are illustrated in the box plots. The Mann-Whitney U test was used to compare group values and p-values are depicted in the panels.

Table 2 summarizes the number, duration and highest DNA-copy number of CMV reactivations in VI and VR patients. The highest DNA-copy number differed significantly between groups, with higher levels in VI, (p = 0.002) and a clear trend towards a difference in infection duration was also observed (p = 0.053). Because all of these parameters (number, duration and DNA-copy number) may affect the production of anti-CMV antibodies, we designed a new variable that consider all of them. We graphed the follow-up time on the x-axis and the number of CMV-DNA copies on the y-axis, and we calculated the CMV-AUC for each patient as a measure of infection intensity. Fig 1 shows the course of the CMV infection in two representative patients, one of them VR and the other VI. Intensity of infection was clearly higher in recently infected patients than in those who experienced reactivations after transplantation (Mann-Whitney U test, p = 0.00002), with no differences in follow-up time and, means of 317.5 (IR: 214 days) and 349.4 days (IR: 171 days), respectively. The four patients with the highest CMV-AUC levels were patients in the VI group.

**Table 2:** Characteristics of CMV infection in VI and VR patients.

	VI*	VR	p
**Number of reactivations**	3 (IR:1.25)	2 (IR:2)	0.09
**Duration (days)**	97 (IR:116)	32.5 (IR:41.75)	0.053
**Highest DNA-copy number**	35,380 (IR:123,320)	1,430 (IR:2010)	0.002

*Including first infection

Seronegative patients undergoing primary CMV infection showed a more prolonged infection with increased viremia compared to reactivations in seropositive patients.

### Evolution of anti-CMV antibody titers during the follow-up period

**Fig 2:**
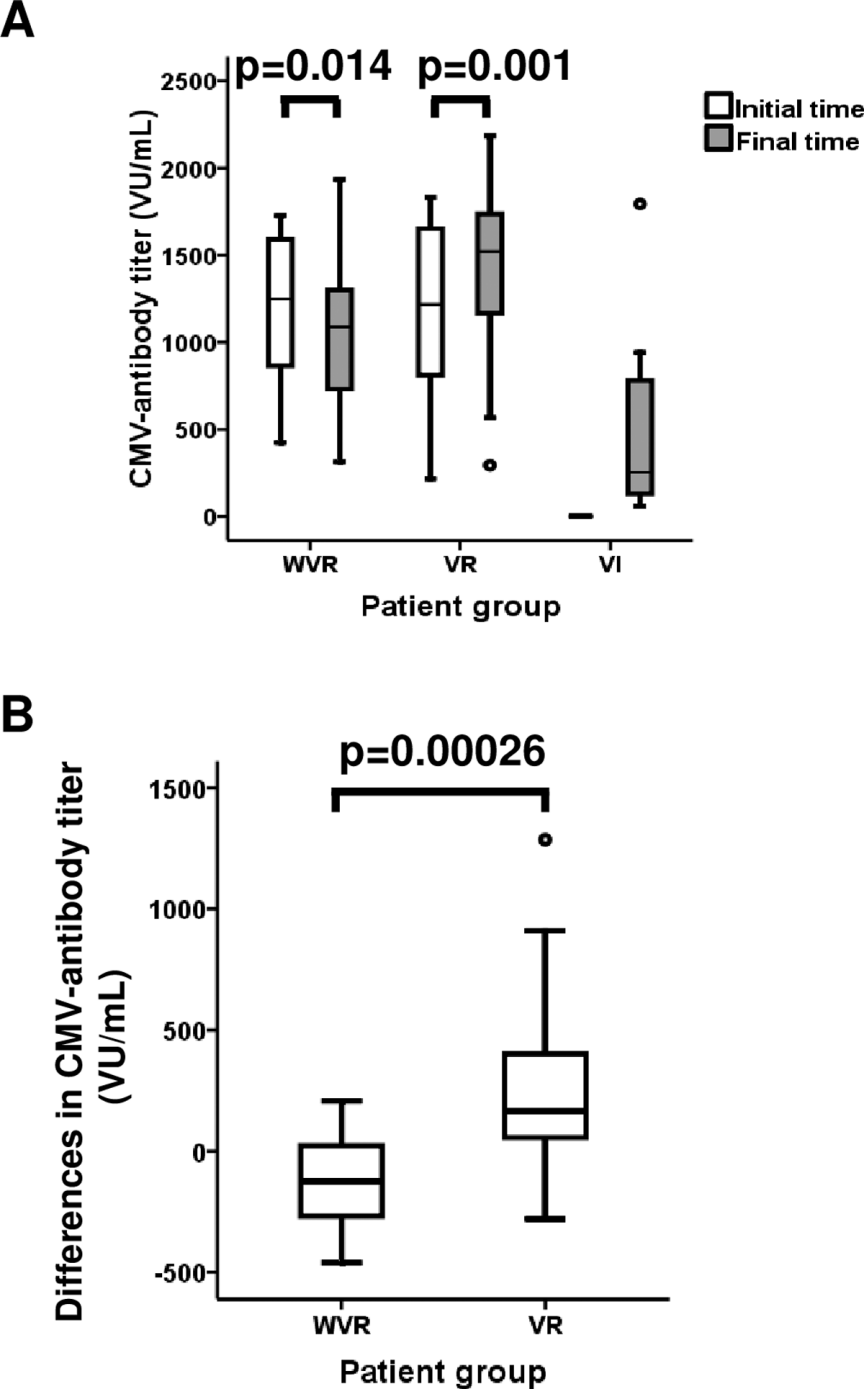
CMV-antibody titers and variations in CMV-antibody titers in kidney transplant patients. CMV antibody titers were quantified by ELISA in the sera of patients prior to transplantation and at the end of follow-up. (A) Comparisons of CMV antibody titers at transplantation and at the end of follow-up in WVR (n = 16), VR (n = 26) and VI (n = 7) transplant patients. A paired t test was used to compare paired means between WVR and VR patients. (B) Variations in CMV antibody titers of WVR and VR patients were compared. Student´s t test was used to compare groups. Outlier values, represented by circles, were calculated by adding 1.5 times the IR to the 75th percentile. Significant differences are indicated with p-values described in the panels.

Titers of anti-CMV antibodies were quantified at the time of transplantation and at the end of the follow-up period. Levels of anti-CMV antibodies in seropositive individuals without virus reactivations (WVR) declined significantly during the follow-up, with means of 1,208 VIRO units (VU)/ml (SD: 437 VU/ml) at transplantation and 1,074 VU/ml (SD: 450 VU/ml) at the end of the follow-up (p = 0.014, Student´s paired T test) (Fig 2A). By contrast, antibody levels increased significantly in VR patients, from 1,174 VU/ml (SD: 498 VU/ml) initially to 1,432 VU/ml (SD: 495 VU/ml) after follow-up (p = 0.001, Student´s paired T test) (Fig 2A). Differences between the two samples were also significant when the two groups of transplant patients, WVR and VR, were compared (p = 0.00026, Student´s T test) (Fig 2B). Levels of anti-CMV antibodies produced by VI patients without antibodies present at transplantation, were heterogeneous, with a median of 253 VU/ml (IR: 825) (Fig 2A). Four of the VI patients had no detectable levels of anti-CMV antibodies, probably due to the immunosuppressive therapy. As these patients could not immunologically control the CMV infection, they showed the highest CMV-AUC values.

**Fig 3:**
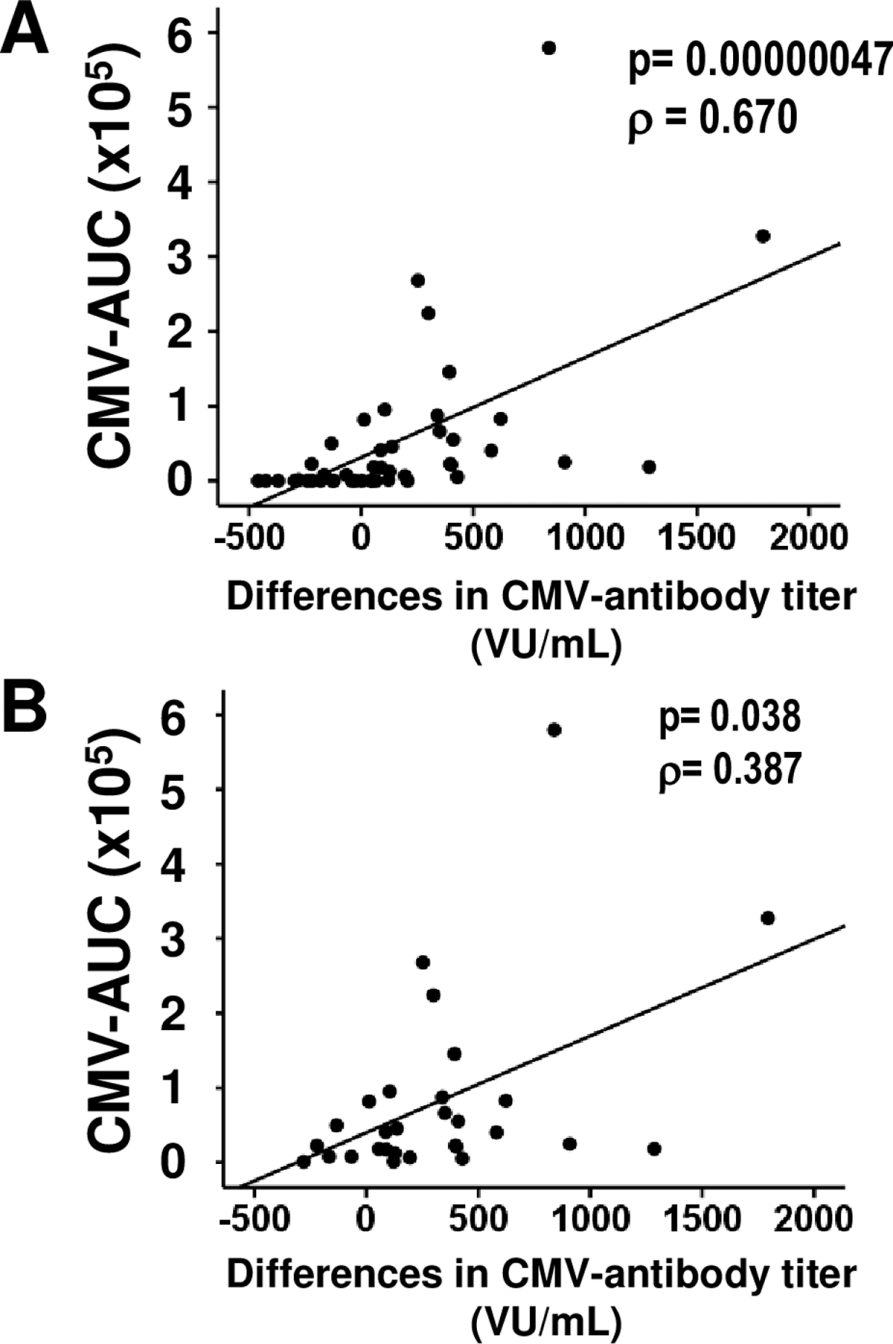
Association between anti-CMV antibody titers and CMV-AUC. Correlations between the difference in anti-CMV antibody titers and CMV-AUC throughout the follow-up period were studied in all transplant patients (A) and only in infected patients (B). Spearman correlation coefficients (ρ) and corresponding p-values are listed in the upper right-hand corner.

Because we are quantifying the intensity of CMV infection by matching the number of CMV DNA copies and the time elapsed since the transplant, by comparing this infection intensity (CMV-AUC) to the variation in the anti-CMV antibody titer in the patients (current titer minus the initial titer), we can get an idea of the effect of the intensity of the reactivation on the levels of anti-CMV antibodies. VI patients with undetectable levels of antibodies were excluded from this analysis. A strong induction of CMV-specific antibodies or a boost in the pre-existing CMV-specific antibody responses during virus reactivation is positively correlated with a strong CMV-AUC, not only when all patients are examined (Spearman test, rho = 0.670, p = 0.00000047) (Fig 3A), but also when we focus only on patients who were seropositive at the time of transplantation (Spearman test, rho = 0.387, p = 0.038) (Fig 3B).

Anti-CMV antibody titers declined after transplantation in WVR patients; however, the titers increased in VR patients. Therefore, both the time the reactivation lasted and the number of DNA copies generated during the reactivation were related to the titer of anti-CMV antibody production.

### Anti-HLA-antibody titers in transplant patients

**Fig 4:**
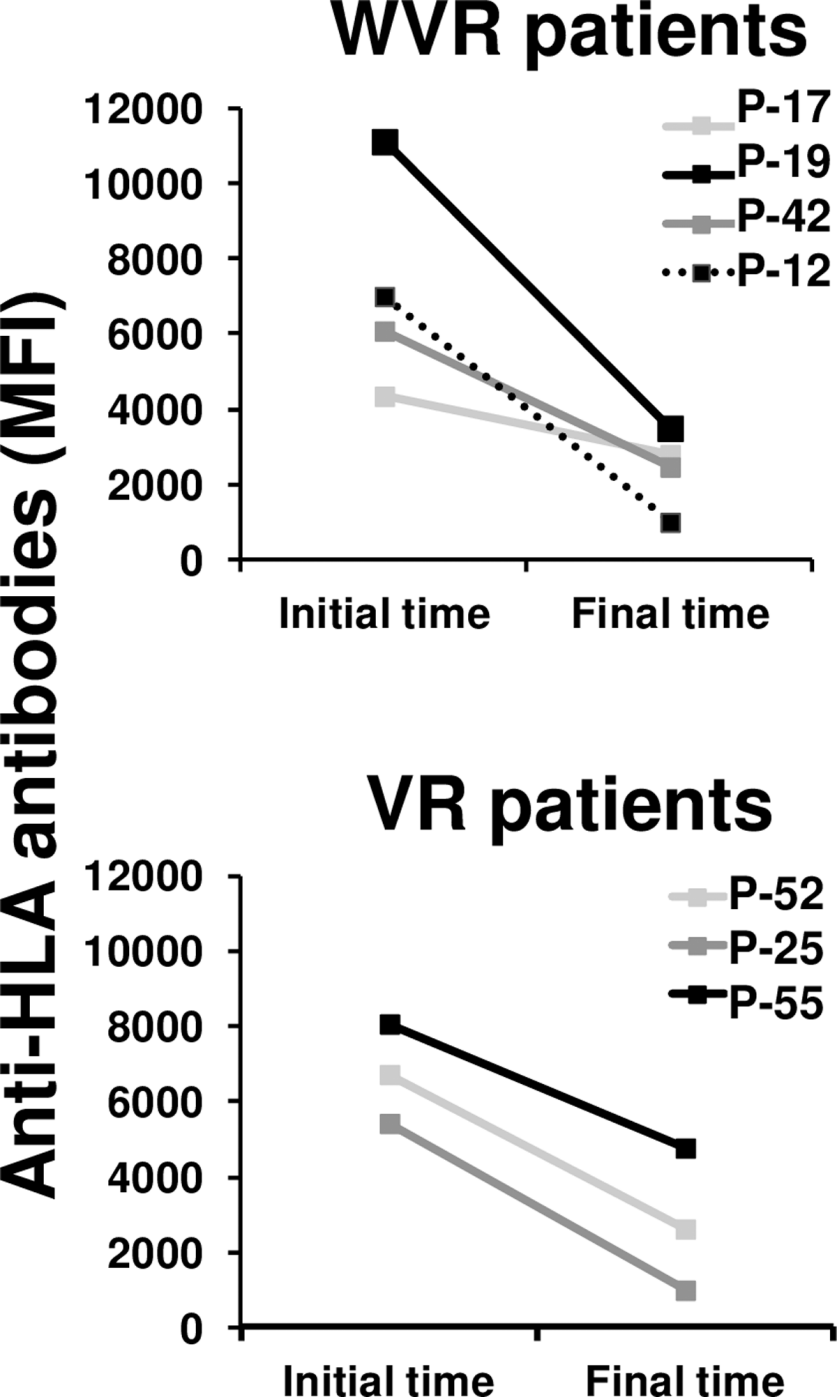
Anti-HLA antibodies in transplant patients. Anti-HLA antibodies were analyzed in kidney transplant patients before transplantation and at the end of follow-up by LABScreen® assays using Luminex xMAP technology. Initial and final measurements of MFI from previously immunized patients with no donor-specific antibodies are represented in the figure (WVR: n = 4 and VR: n = 3).

After confirming that there was a decrease in the anti-CMV antibody titers in WVR patients, probably produced by the immunosuppression in the transplanted individuals, we wanted to know whether this decrease was specific for anti-CMV antibodies or whether it also occurred in other antibodies against antigens that did not affect the patients' immune systems. We analyzed the presence and levels of anti-HLA antibodies in the patients prior to kidney transplantation. Only seven patients were HLA-immunized at that initial time (VR, n = 3; WVR, n = 4). None of the patients developed donor-specific anti-HLA antibodies, and all of them showed diminished levels of antibodies at the end of the follow-up time, regardless of whether they belonged to the VR or WVR groups (Fig 4).

These results suggest that the increase in anti-CMV antibody titers is a specific effect in response to virus reactivations.

### Anti-CMV antibody titers and degree of T-lymphocyte differentiation

**Fig 5:**
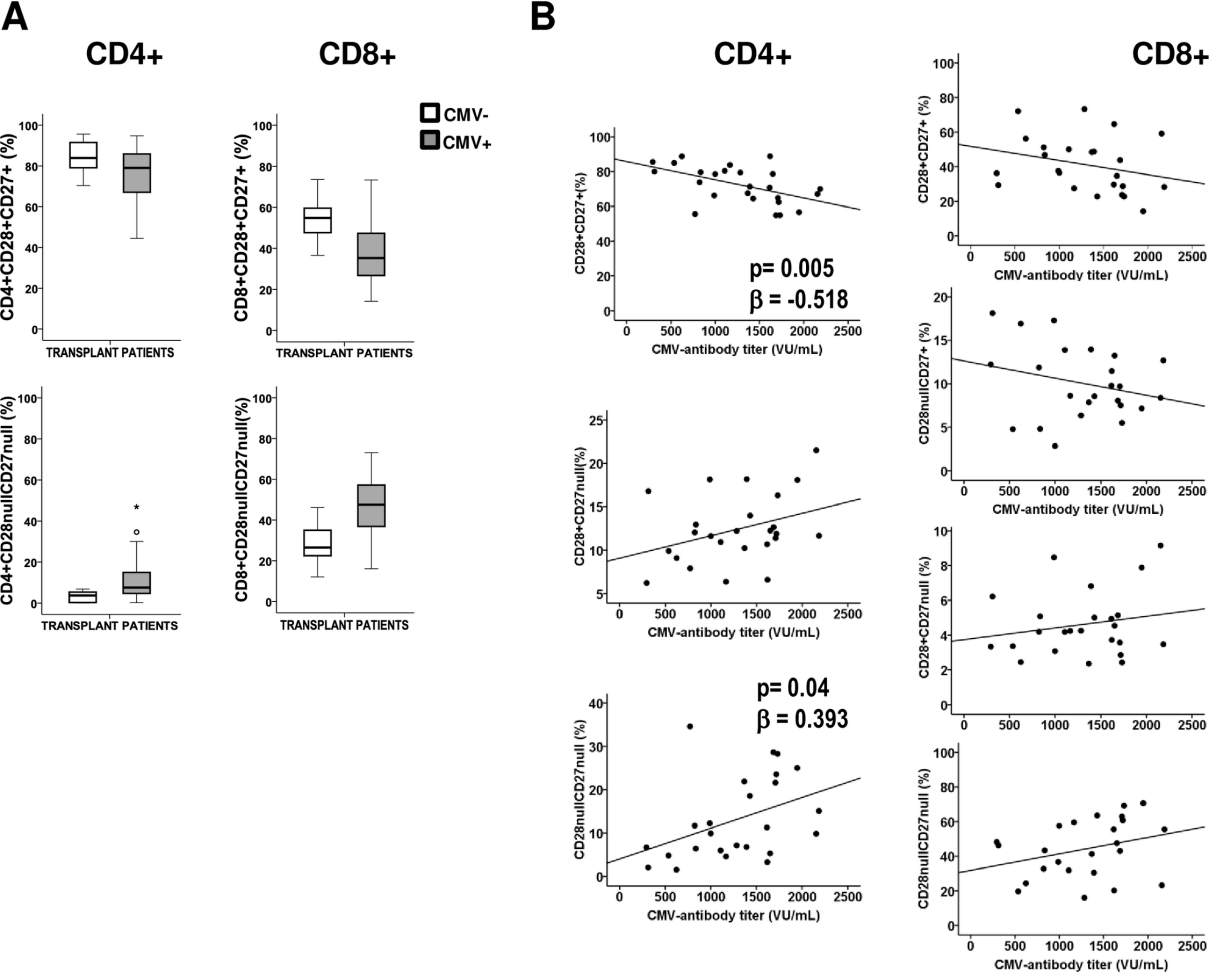
Differentiation status of CD4+ and CD8+ T-lymphocytes according to CMV serostatus and anti-CMV antibody levels. (A) Distribution of CD4+ and CD8+ T-cells into CD28+CD27+ (less-differentiated) and CD28^null^CD27^null^ (more-differentiated) subsets according to patients CMV serostatus. (B) Distribution of CD4+ and CD8+ T-cells into CD28+CD27+ (less-differentiated), CD28+CD27^null^/CD28^null^CD27+ (intermediate), and CD28^null^CD27^null^ (more-differentiated) subsets was related to anti-CMV antibody titers in CMV-seropositive patients (n = 24). Expression of CD28+ and CD27+ was analyzed by flow cytometry in gated CD3+CD4+ T-cells and in CD3+CD8+ T-cells. Linear regression coefficients adjusted by age and corresponding p-values are indicated.

We previously demonstrated that anti-CMV antibody titers show a positive correlation with the T-lymphocyte differentiation status in elderly people [25]. We wanted to verify this association in our studied group of transplant patients because, in these patients, the intensity of the CMV-infection correlates with antibody production against the virus. We classified T-lymphocytes into functionally distinct populations using a combination of CD27 and CD28 markers. These surface markers allowed us to distinguish between less-differentiated (CD28+CD27+), intermediate (CD28+CD27^null^ or CD28^null^CD27+, the last only in CD8+), and more-differentiated (CD28^null^CD27^null^) cells [26]. First, we compared the distributions of the T-cell subpopulations in seropositive (n = 24) and seronegative (n = 6) transplant patients, none of whom had received induction treatment based on T-lymphocyte depletion. These agents work by causing T-cell lysis or clearance, resulting in the depletion of circulating lymphocytes. As described in healthy donors, CMV seropositivity was related to lower frequencies of less differentiated subsets (CD27+CD28+) and to higher frequencies of more differentiated subsets (CD27^null^CD28^null^) of CD4+ and CD8+ T-lymphocytes. Differences in these subsets between CMV-seropositive and CMV-seronegative patients showed a clear trend; however, they did not reach significance, probably due to the low number of seronegative patients (Fig 5A). We then calculated the correlation between the anti-CMV antibody titer and the frequency of these T-lymphocyte subsets. We were unable to demonstrate any associations in CD8+ T-lymphocytes (Fig 5). Nevertheless, an effect was evident in CD4+ T-lymphocytes, where CD27+CD28+ showed an inverse correlation (linear regression, p = 0.005) and CD27^null^CD28^null^ showed a positive correlation (linear regression, p = 0.04), adjusted by age (Fig 5B), with the anti-CMV antibody titers.

These results demonstrate that transplant patients with higher anti-CMV antibodies titers showed a more differentiated CD4+ T-lymphocyte status.

## Discussion

CMV infection has harmful effects on the immune system and overall health, but the reasons behind its heterogeneous impact on individuals are not well understood. We found that virus replication is more aggressive in patients who are infected after transplantation, and that some patients did not produce detectable levels of anti-CMV antibodies. These patients have a substantially higher viral load and prolonged viremia in a primary infection compared to re-infection. Patients with virus reactivations produced anti-CMV antibodies, whose levels correlated with infection intensity, quantified as the number and duration of reactivations and the number of CMV-DNA copies. By contrast, patients without virus reactivations displayed a reduction in their antibody levels after transplantation, similar to the levels of antibodies against other specificities, such as anti-HLA antibodies. As previously demonstrated in other studied groups, a higher degree of differentiation of CD4+ T-lymphocytes also correlated positively with antibody levels.

Titers of anti-CMV antibodies may be indicative of the history of infection, and they could be used as a measure to quantify the interaction of the immune system with the virus, including time since infection and the severity or frequency of reactivations. Reactivation can be detected in response to inflammation, stress or immunosuppression [1, 2], and it is more frequent in the elderly and other immunosuppressed individuals. In fact, elderly people have higher levels of antibodies than young individuals, and these levels have been positively associated with the frequencies of specific anti-CMV CD4+ T-lymphocytes [25]. Transplant patients suffer a high number of CMV-reactivations, because they receive immunosuppressive treatment in order to avoid rejection of the transplanted organ. It would be necessary to study healthy individuals over many years to detect as many reactivations as we could find in our group of transplant patients. Thus, in a 12-year follow-up of a cohort of older women, both serostatus and CMV-DNA detection remained the same at baseline and at follow-up [27]. Moreover, in kidney transplant patients, according to consensus guidelines on CMV management [28], the course of infection is monitored periodically in order to detect virus reactivations, treat patients, and control viral replication as soon as possible. This information allowed us to have an accurate idea of the status of the infection during the follow-up period.

CMV infection has been linked to a variety of chronic diseases with an inflammatory component, including cardiovascular diseases, cancer and cognitive and functional impairment [20]. CMV infection is also closely related to immunosenescence, characterized by a clonal T-lymphocyte expansion, associated with a lower diversity of the TCR repertoire. This has been proposed as an explanation for much of the decreased ability of the elderly to resist new infections and respond effectively to reinfection and persistent infections [29, 30]. In kidney transplant patients, primary CMV infection has been shown to have a substantial impact on the differentiated memory phenotype of peripheral T-lymphocytes, and it may also negatively affect renal allograft function [31]. Additionally, many differences can be found in both the T-lymphocyte differentiation and clinical status of individuals infected with CMV. These differences have led to efforts to identify other factors that make it possible to classify different risk profiles depending on the course of infection throughout the individual's life. One of these proposed parameters consists of the anti-CMV antibody titers. In several studies conducted in elderly women and Latinos, a high association has been found between their fragility, disability, and mortality and the anti-CMV IgG titers. [17, 32]. Other studies have also reported an association between levels of anti-CMV antibodies and increased mortality risk, partly through their relationship with cardiovascular disease [16–20]. Recently, we demonstrated that anti-CMV antibody levels are related to the T-cell differentiation status and T-cell immunocompetence in the elderly [15, 25]. However, other authors have failed to find this association [33–35]. The detection of CMV-DNA has been proposed as a means to monitor chronic CMV infection, independently of the time since the initial infection or the possible reactivations of the virus over time. However, the influence on the immune system and, for instance, on the ability of the CMV virus to be activated and cause active disease, may be the result of the history of infection, and anti-CMV antibody levels may be a reflection of all the events of prior exposure to the virus. We would expect greater exposure of the immune system to the virus through higher and more prolonged viremia to lead to higher antibody titers.

The study of transplant recipients also has some limitations. Because patients are pharmacologically immunosuppressed, their immunological responses are defective and may not reflect the situation of a healthy individual. To avoid organ rejection, treatment with immunosuppressants, including calcineurin inhibitors, inhibits both naïve and memory T-lymphocyte activation by blocking downstream signaling of the TCR [36]. This mechanism effectively suppresses alloimmunity in transplant recipients, but it also impairs responses to CMV infection or reactivations [37, 38]. Memory immunological responses were more preserved in patients who were already infected before receiving the transplant. Whereas some of the patients infected after kidney transplantation were not able to generate anti-CMV antibodies at detectable levels, in previously infected patients, the production of antibodies correlated with the frequency and intensity of the reactivations. However, one of the effects of immunosuppression may be a reduction in the antibody titers, as we demonstrated for the levels of anti-CMV in WVR patients and anti-HLA antibodies in previously HLA-immunized patients, after the transplant and immunosuppressive treatment.

In summary, our data show that titers of anti-CMV antibodies are related to the course of CMV infection in kidney transplant patients, and positively correlated with the degree of CD4+ T-lymphocyte differentiation. Levels of specific antibodies might be useful as a marker of the history of CMV infection and, therefore, of immunocompetence throughout the individual’s life.
